# Early Life History of Deep-Water Gorgonian Corals May Limit Their Abundance

**DOI:** 10.1371/journal.pone.0065394

**Published:** 2013-06-10

**Authors:** Myriam Lacharité, Anna Metaxas

**Affiliations:** Department of Oceanography, Dalhousie University, Halifax, Nova Scotia, Canada; Heriot-Watt University, United Kingdom

## Abstract

Deep-water gorgonian corals are long-lived organisms found worldwide off continental margins and seamounts, usually occurring at depths of ∼200–1,000 m. Most corals undergo sexual reproduction by releasing a planktonic larval stage that disperses; however, recruitment rates and the environmental and biological factors influencing recruitment in deep-sea species are poorly known. Here, we present results from a 4-year field experiment conducted in the Gulf of Maine (northwest Atlantic) at depths >650 m that document recruitment for 2 species of deep-water gorgonian corals, *Primnoa resedaeformis* and *Paragorgia arborea*. The abundance of *P. resedaeformis* recruits was high, and influenced by the structural complexity of the recipient habitat, but very few recruits of *P. arborea* were found. We suggest that divergent reproductive modes (*P. resedaeformis* as a broadcast spawner and *P. arborea* as a brooder) may explain this pattern. Despite the high recruitment of *P. resedaeformis*, severe mortality early on in the benthic stage of this species may limit the abundance of adult colonies. Most recruits of this species (∼80%) were at the primary polyp stage, and less than 1% of recruits were at stage of 4 polyps or more. We propose that biological disturbance, possibly by the presence of suspension-feeding brittle stars, and limited food supply in the deep sea may cause this mortality. Our findings reinforce the vulnerability of these corals to anthropogenic disturbances, such as trawling with mobile gear, and the importance of incorporating knowledge on processes during the early life history stages in conservation decisions.

## Introduction

Deep-water gorgonian corals (Octocorallia: Alcyonacea; “sea fans”) are increasingly being recognized as important foundation species of deep-sea benthic ecosystems, particularly on seamounts and continental margins [1]. With their typical arborescent shape, these organisms form structures, which foster biodiversity, either as substrate for epifaunal communities [2]–[4], or by creating shelter for bottom-dwelling fish from strong currents and predators [5], [6]. The protruding shape of the corals, however, along with slow growth and high longevity [7], [8], make them vulnerable to destructive fishing practices, such as trawling with mobile gear [9]–[11]. Most deep-water gorgonians require hard substrate for settlement and, as suspension-feeders, strong currents for the delivery of food. Hence, they typically are found on steep topographic features on shelf breaks and the upper continental slope [12]–[16], where strong bottom currents prevent the accumulation of fine-grained sediment and increase the encounter rate with food particles [17].

The reproductive ecology of deep-water corals (including gorgonians) remains largely undescribed. Corals undergo sexual reproduction to disperse to new habitats. Colonies are either male or female (gonochorism), or both (hermaphroditism), and produce planula larvae through 2 reproductive modes: fertilization inside or on the surface of the female colony (brooding) or external fertilization where gametes from both male and females colonies are released in the water column (broadcast spawning) [18]. In octocorals (including shallow-water species), gonochorism dominates, but broadcast spawning and brooding are equally present among species [19]. Planula larvae subsequently disperse as plankton before eventually settling on a suitable substrate (settlement) and metamorphosing into a benthic stage. Measures of recruitment reflect our ability to detect the primary polyp stage after a certain period of time. Physical factors influencing recruitment (or post-settlement mortality) have not been assessed for deep-water gorgonian corals. For sessile organisms, these factors include predation, disturbance (physical or biological) and space and/or resource competition [20]. Once settled, corals undergo asexual reproduction when additional polyps bud from the primary polyp to form a juvenile colony.

Despite its importance in determining population dynamics, our knowledge of the magnitude, frequency and environmental factors influencing recruitment of deep-sea (>200 m) benthic invertebrates is incomplete. The presumed environmental stability of deep-sea ecosystems, in particular on the abyssal plains, led to the hypothesis that most deep-sea organisms grow slowly, mature late and live to a greater age, while investing relatively less energy into reproduction than their shallow-water counterparts [21]. Consequently, the recruitment rate is expected to be low, given the investment in growth and maintenance, rather than in the colonization of new habitats [22], [23]. However, fast recruitment rates have been recorded on artificial food patches [24], and in unstable and transient deep-sea environments, such as hydrothermal vents [25], [26] and whale falls [27], where colonization rates tend to vary spatially and temporally. Continental margins, the transitional habitats between the shallow continental shelves and deep abyssal plains, are now considered to harbour dynamic, heterogeneous ecosystems increasingly affected by anthropogenic disturbances [28]. Knowledge is lacking on the reproductive strategies of organisms of the diverse epibenthic megafauna inhabiting continental margins, in particular cnidarians and poriferans.

To our knowledge, in-situ measures of recruitment of deep-water corals are not available in waters deeper than 200 m, where these organisms are most commonly found [1]. At these depths, recruitment has been inferred only sporadically by analysing size-frequency distributions of adult colonies. Off the Hawaiian Archipelago (375–450 m), slow recruitment, slow growth, and high mortality during the early benthic life stages have been suggested to limit the population of the gorgonian *Corallium secundum* (pink coral) [29], while the periodicity of recruitment events of the solitary scleractinian *Desmophyllum dianthus* is estimated at 25 years off the Tasmanian coast (Australia; 1,000–2,100 m) and adjacent seamounts [30]. At depths <200 m, size-frequency distributions indicated that the cosmopolitan scleractinian *Lophelia pertusa* recruited annually on oil platforms in the North Sea, and the magnitude of these recruitment pulses decreased with increasing distance from the potential source population located 10 s of km’s away, off the northern coast of Scotland [31].

In Atlantic Canada, fishermen have long associated gorgonians, referred to as the ‘trees’, with highly productive fishing grounds [32], in different locations along the continental margin, including the deep Northeast Channel, which separates Georges Bank and the Scotian Shelf in the Gulf of Maine. Northeast Channel harbours the highest known density in Atlantic Canada of intact colonies of 2 species of large gorgonians: the seacorn coral *Primnoa resedaeformis*, and the bubblegum coral *Paragorgia arborea* [14]. A coral conservation area (424 km^2^) was established by Fisheries and Oceans Canada in 2002 at the shelf-edge of Northeast Channel, extending to a depth of 1,200 m to prevent further damage. In the Northeast Channel Coral Conservation Area, *P. resedaeformis* and *P. arborea* have aggregated distributions [13], [33], and given the recurrent observations of uncolonized substrate within these regions, the availability of suitable substrate is not thought to limit their distribution [13], [33]. Few small colonies (<10 cm) have been observed, leading to the hypothesis that reproductive processes may be limiting these populations [33].

The preferred substrate of *P. resedaeformis* and *P. arborea* is a mixture of cobbles, pebbles and boulders [13], [33]. Such hard substrate is necessary for their settlement, even in areas of moderate relief [15], underscoring the potential influence of microhabitat complexity (three-dimensional) on post-settlement processes. Habitat complexity increases the surface area available for settlement and provides shelter from physical and biological disturbances [34]. As trawling with mobile gear has been shown to alter benthic habitat complexity [35], this characteristic of the recipient habitat could be critical in the potential of deep-water corals to recover from anthropogenic disturbance.

Here, we report results of a 4-year field experiment in the Northeast Channel Coral Conservation Area where we determined: 1) the magnitude of recruitment, and 2) the role of substrate complexity in recruitment for the deep-water gorgonian corals *P. resedaeformis* and *P. arborea*. We found that recruitment was high for *P. resedaeformis*, but limited for *P. arborea*. We suggest that this difference possibly indicates divergent reproductive modes in these species. For *P. resedaeformis*, the 3-dimensional structural complexity of the recipient environment influenced recruitment. However, despite high larval supply and recruitment, we suggest that mortality occurring after settlement is particularly significant in determining the recruitment rate in this species, and hence the abundance of larger, adult colonies.

## Materials and Methods

### Study Area & Sites

The Northeast Channel, separating Georges Bank and the Scotian Shelf, is the only deep passage connecting the northwest Atlantic with the Gulf of Maine proper [36]. Water circulation in the channel is dominated by tides, with an inflow alternating between Warm Slope Water and Labrador Slope Water along the northeastern side, and outflow of Maine Intermediate Water along the southwestern side [36]. At the shelf edge, where the channel is 20–30 km wide, the seafloor plunges from a depth of 210–370 m into 3 steep-walled submarine canyons to a maximum depth of ∼1,000 m [36], [37]. The detailed water circulation within these canyons is unknown. The field experiment was conducted at 3 locations in the Middle Canyon of Northeast Channel Coral Conservation Area. Two locations were on opposite walls of the canyon separated by a distance of ∼2 km (north wall – depth: 658 m; south wall – depth: 671 m), while the third location was on the floor of the canyon (floor – depth: 863 m). Permission to perform this field experiment was granted by Fisheries and Oceans Canada.

The surficial geology of the Northeast Channel and adjacent upper continental slope is a relic of the glacial history of the region, being mostly comprised of ice-contact sediment such as pebbles, cobbles and boulders in a matrix of coarse sand patches [38]. The seafloor is swept by strong currents, which prevent the accumulation of fine-grained sediment [17], [38]. Large gorgonian corals are often observed on the tops and sides of boulders [13], [33], [38]. On the north and south walls sites of the Middle Canyon, we observed a similar surficial geology, while on the floor of the canyon, the seafloor was mostly comprised of sand and sparse cobbles and pebbles, but a large boulder (>3 m in width) was present within 5 m of the larval settlement collectors (see Experimental design below).

The local abundance of coral colonies differed between the canyon locations where the experiment was conducted. On the north wall of the canyon, we observed multiple thickets of large, dense *P. resedaeformis* colonies within 10 m of the collectors (the closest colony was approximately 1 m away from the collectors). We did not observe colonies of *P. arborea* within meters of the collectors, but colonies were present within 100 m. On the south wall of the canyon, a single thicket of *P. resedaeformis* and a *P. arborea* colony were observed within 10 m of the collectors. At the floor of the canyon, a large colony of *P. arborea* (>1.5 m in height and width) and few smaller colonies of the same species were present within 5 m of the collectors, and more were present within 10 s of meters. Colonies of *P. resedaeformis* were not observed at this location.

**Figure 1:**
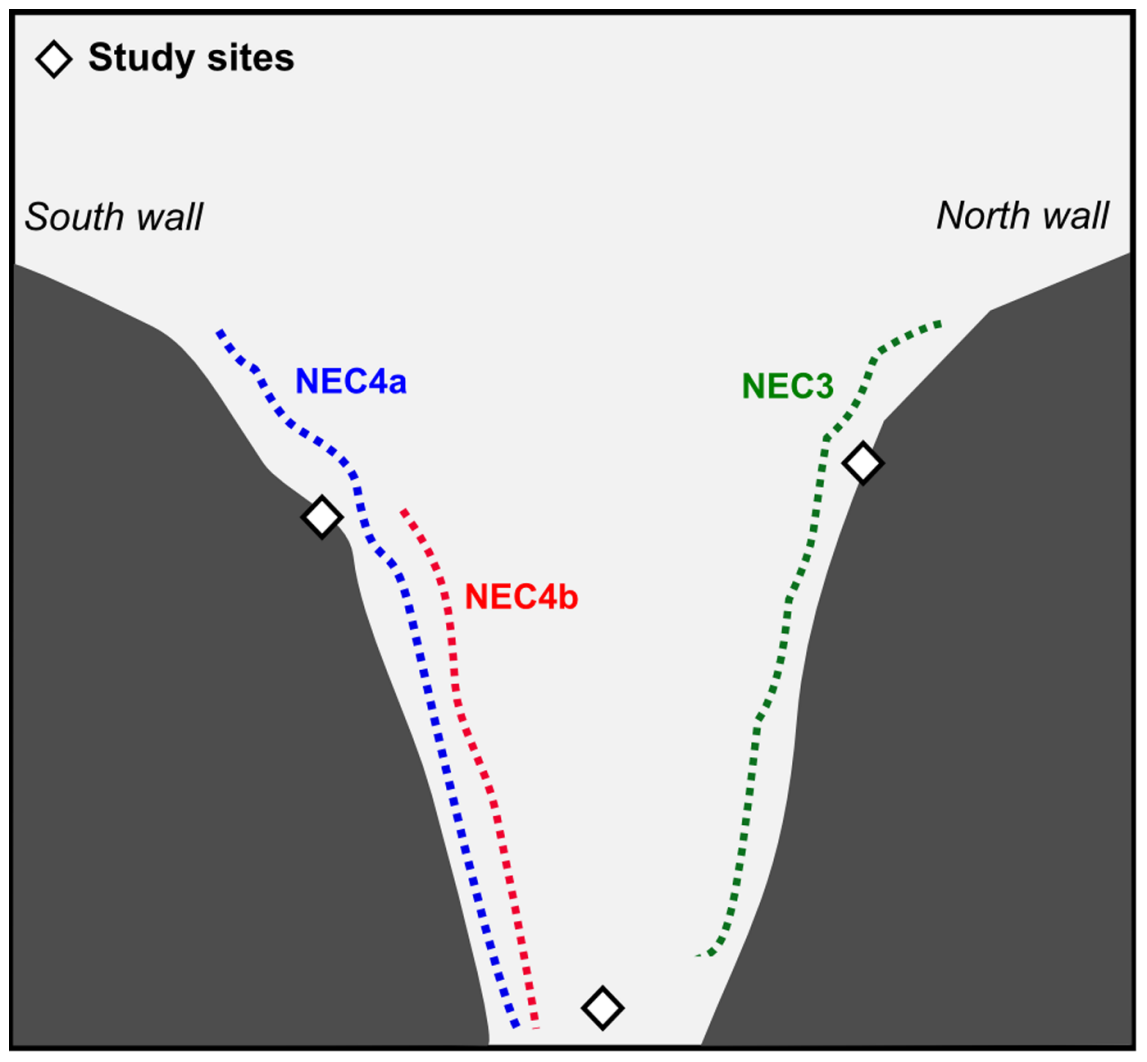
Study sites (this study) and locations of abundance transects [33]. Experimental study sites in the Middle Canyon of the Northeast Channel Coral Conservation Area (Gulf of Maine). The sites are shown relative to 3 upslope transects (‘NEC3’, ‘NEC4a’, ‘NEC4b’) performed in the area in 2006 [33]. Diagram is not to scale.

Three video transects were performed in the area when the arrays of larval settlement collectors were deployed [33] (Fig. 1). A summary of the mean and maximum abundance of *P. resedaeformis* and *P. arborea* is presented in Table 1. In all transects, the density of *P. resedaeformis* decreased with increasing depth. On average, less than 1 colony/10 m^2^ was observed at depths greater than 750 m. No relationship was observed between the density of *P. arborea* colonies and depth, although higher densities tended to be observed at greater depths than *P. resedeaformis* [33]. This pattern has also been reported on the continental margins of Newfoundland [16] and Norway [15].

**Table 1:** Summary of mean and maximum abundance of *P. resedaeformis* and *P. arborea* along 3 transects in the Middle Canyon of the Northeast Channel Coral Conservation Area [33].

	Canyon location (Transect)	Mean abundance ± SD, n (10 m^−2^)	Maximum abundance (10 m^−2^)	Depth interval of maximum abundance (m)
***P. resedaeformis***	North wall (NEC3)	2.37±5.52, 263	37.7	500–525
	South wall (NEC4a)	1.30±1.54, 280	18.3	600–625
	South wall (NEC4b)	3.08±4.26, 146	8.96	675–700
***P. arborea***	North wall (NEC3)	0.09±0.54, 263	5.88	725.750
	South wall (NEC4a)	0.44±1.63, 280	15.0	825–850
	South wall (NEC4b)	1.08±1.54, 146	10.0	850–875

### Experimental Design

**Figure 2:**
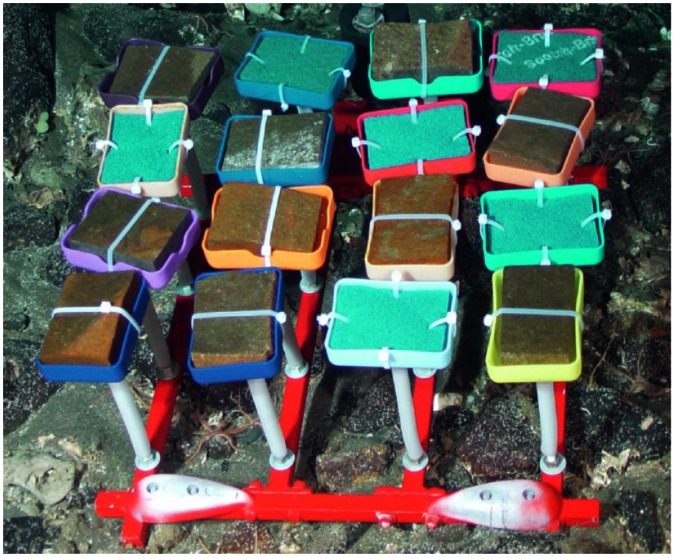
Array of larval settlement collectors at deployment in 2006 (South wall: 671 m). Collectors included either a basalt rock (∼7×10×2 cm) supported by a plastic ring (n = 10), or mesh pads (∼7.5×10×1.5 cm, blue ‘Scotch-Brite’ pads, mesh openings: ∼3.5 mm^2^) (n = 6).

Recruitment was measured between July 2006 and August 2010. Because of the longevity of deep-water corals (spanning 10 s to 100 s of years), and unknown periodicity of reproductive dynamics, this deployment period was assumed to be sufficient to capture and integrate early life history processes. Sixteen larval settlement collectors were attached to a single galvanized steel frame for ease of deployment with a 20-cm steel threaded rod covered with a plastic tube (Fig. 2). Collectors included either 1) a basalt rock (∼7×10×2 cm) supported by a plastic ring (n = 10), or 2) mesh pads (∼7.5×10×1.5 cm, ‘Scotch-Brite’ pads, mesh openings: ∼3.5 mm^2^) (n = 6). More basalt rocks than mesh pads were included because 1) hard substrate is most prevalent in this area, and 2) it is the preferred substrate of the species of interest. Each settlement plate was placed in a plastic container pierced with holes on the underside to allow water flow, which is critical for the recruitment of suspension-feeders. The experimental array of larval settlement collectors may have influenced the small-scale flow velocity, but we considered that its structure reasonably mimicked the surrounding seafloor habitat in the Middle Canyon. The individual collectors were randomly positioned on the frame, and separated by a few centimeters, therefore representing independent samples, particularly for sedentary colonists. The total surface area available for recruitment on the collectors was 547 cm^2^ for those composed of basalt rocks (top planar surface area: 70 cm^2^) and 322 cm^2^ for those composed of mesh pads (top planar surface area: 79 cm^2^). Only the top planar surface of the mesh pads was considered suitable for settlement since deep-water gorgonian corals are suspension-feeders that require access to water flow. Arrays of collectors were deployed and recovered at each location in a polycarbonate lidded box (∼80×60×35 cm) with the remotely-operated vehicle ROPOS. To avoid dislodgement of organisms during ascent, a piece of open cell foam (∼ 5 cm in thickness) was attached to the underside of the box lid. All components of the collectors and the corresponding section of the foam were preserved in 95% ethanol at sea.

### Sample Processing

**Figure 3:**
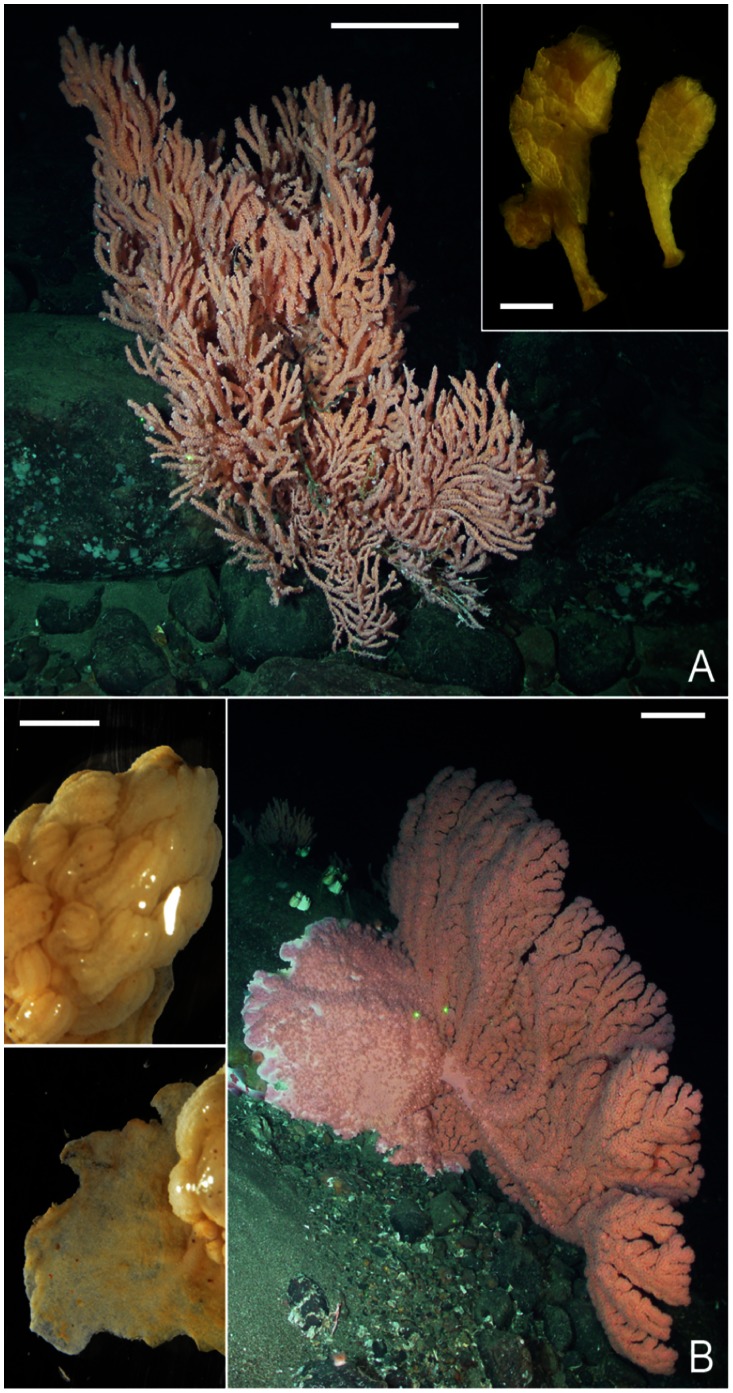
Recruits and adult colonies of deep-water gorgonian coral in Northeast Channel. Scale bars for adult colonies represent 20 cm. (A) *P. resedaeformis*. Depth: 288 m. Scale bar in inset represents 1 mm. (B) *P. arborea*. Depth: 314 m. Scale bar in inset represents 5 mm.

Coral recruits were identified morphologically based on high-resolution pictures of adult colonies. We used available genetic information to confirm the identity of *P. resedaeformis*, but genetic identification of *P. arborea* was inconclusive given the few recruits we retrieved (see Results). Therefore, we used distinct morphological features to distinguish *P. arborea* recruits from those of *P. resedaeformis* recruits. We recorded the abundance of recruits on the basalt rocks and mesh pads, the plastic tube around the rod, the plastic container, and the plastic ring. For the basalt rocks, the position of the corals (top vs. underside/sides) was recorded. Corals retrieved from foam sections were considered to have dislodged from the top of the settlement plates. Recruits of *P. resedaeformis* were attached to the substrate with a thin basal foot (Fig. 3A - inset). Because of their elongated shape (growing as ‘small trees’), we measured the height with an ocular micrometer mounted on a dissecting microscope (Nikon SMZ1500) from the tip of the foot to the tallest end, and recorded the amount of polyps. Recruits were not measured if the basal foot had been truncated during manipulations. For *P. arborea*, due to its morphology (Fig. 3B – inset), we only recorded the number of polyps, as heights measurements were impractical.

### Statistical Analyses

To determine whether the abundance of *P. resedaeformis* recruits (per 100 cm^2^) differed among canyon locations (north wall, south wall, floor) and types of surfaces on top of the collectors (basalt rocks vs. mesh pads), we performed a 2-way ANOVA with unequal replication with canyon location (3 levels) and surface type (2 levels) as fixed factors. For canyon locations, differences detected with ANOVA were further tested with Tukey’s Honestly Significant Difference (HSD) post-hoc tests. To determine whether 3-dimensional structural complexity of the collectors influenced the abundance of recruits (per 100 cm^2^), we divided the collectors into 2 microhabitats: 1) flat surface on top of the collectors, 2) other components of the collectors (plastic container, plastic ring, plastic tube and sides/undersides of basalt rocks). We performed paired *t*-tests between these microhabitats at each canyon location and for each type of collector. The abundance of recruits (per 100 cm^2^) was square-root transformed to meet assumptions of normality and homoscedacity. To compare the height-frequency distributions of recruits between and within (between types of collectors) locations, we performed 2-sample Kolmogorov-Smirnov (K-S) tests. These tests are robust against deviations from normality and unequal sample size. Statistical analyses were performed in the R programming environment, version 2.14.1.

## Results

We collected recruits at the stages of both primary polyp only and juvenile colonies (2 polyps or more) of the deep-water gorgonian corals *P. resedaeformis* (Fig. 3A) and *P. arborea* (Fig. 3B) on all components of both types (basalt rocks and mesh pads) of collectors. Recruits of both species were collected at each location (north wall, south wall and floor of the canyon), but those of *P. resedaeformis* were far more abundant (Table 2). We retrieved 2 *P. arborea* recruits at the deepest location (floor: 863 m), one of which was composed of more than 20 polyps, the largest recruit retrieved in our study (Fig. 3B – inset). Based on similar morphological features, 2 more recruits were identified as *P. arborea*, one at each location on the north and south walls. The relatively low abundance of *P. arborea* restricted further analyses on this species.

**Table 2:** Total abundance of recruits of the deep-water gorgonian corals *P. resedaeformis* and *P. arborea* retrieved from arrays of larval settlement collectors deployed in the Middle Canyon of the Northeast Channel Coral Conservation Area from 2006 to 2010.

Canyon location	Depth (m)	*P. resedaeformis*	*P. arborea*
North wall	658	1289	1
South wall	671	792	1
Floor	863	97	2

**Figure 4:**
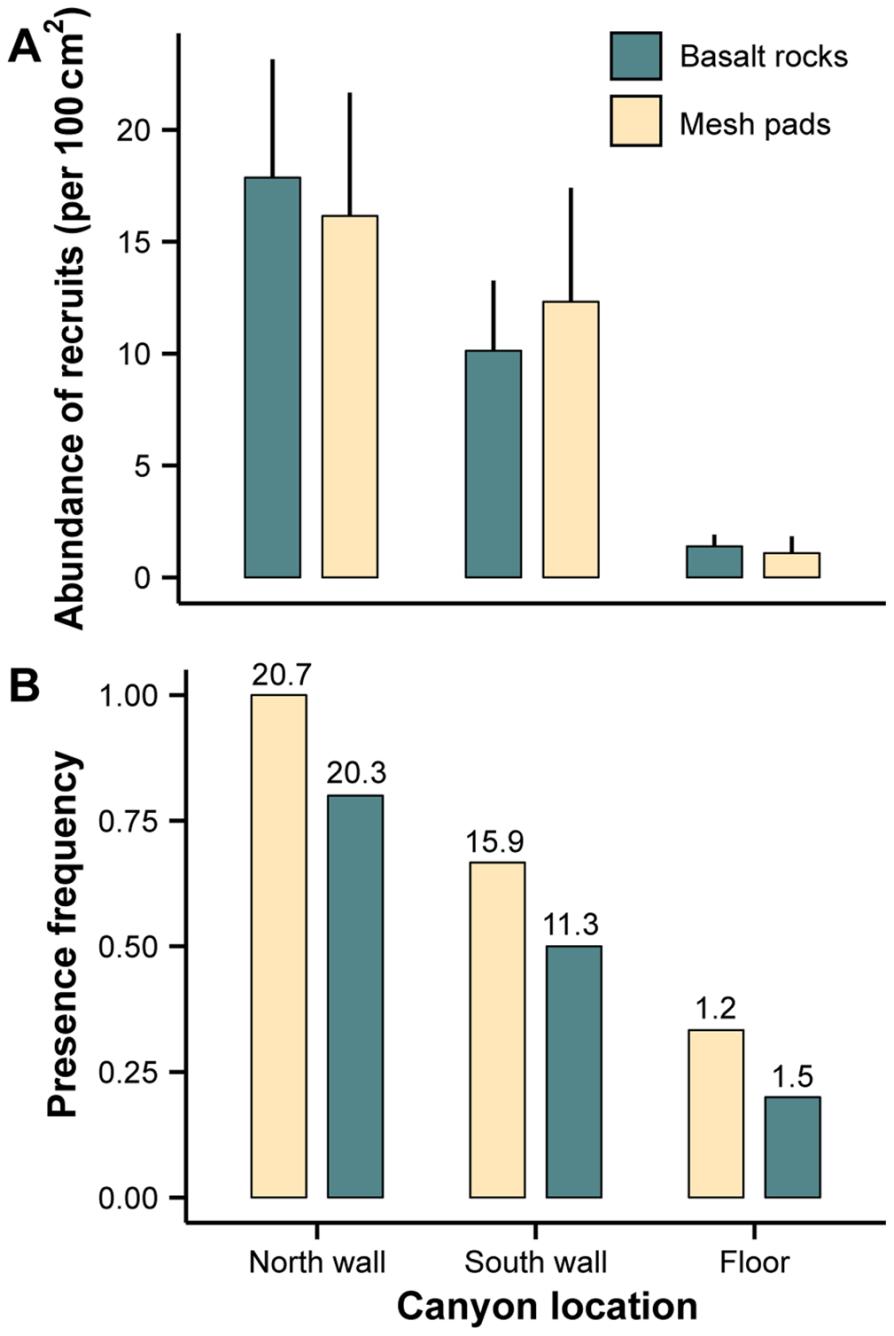
Recruitment of *P. resedaeformis* on arrays of larval settlement collectors. Arrays were deployed at 3 locations (north wall: 658 m, south wall: 671 m, floor: 863 m). Settlement collectors included either a basalt rock (n = 10 per location) or mesh pads (n = 6 per location). (A) Mean abundance (+SD) of recruits on all components of the settlement collectors combined. (B) Frequency of presence of recruits on the top flat surfaces of the larval settlement collectors. Mean abundance of recruits (per 100 cm^2^) on the other components of the settlement collectors (i.e. excluding the top flat surfaces) is indicated at each location and for each surface type.

Total abundance of *P. resedaeformis* recruits (standardized per 100 cm^2^) on the collectors (all components combined) differed among canyon locations, but did not differ between surface types (Fig. 4A) (2-way ANOVA with unequal replication; Location: *F*_2,42_ = 117.09, *P*<0.001, Surface type: *F*_1,42_ = 0.08, *P* = 0.78, Location×Surface Type: *F*_2,42_ = 1.08, *P* = 0.35). Total abundance of recruits differed among all 3 canyon locations (Tukey’s HSD, *P*<0.001), being highest on the north wall (17.22± SD: 5.26 recruits/100 cm^2^), intermediate on the south wall (10.95± SD: 3.97 recruits/100 cm^2^), and lowest at the floor of the canyon (1.28±0.62 recruits/100 cm^2^).

We found few *P. resedaeformis* recruits on the flat top surfaces of the basalt rocks and mesh pads (no recruitment on 21 of the 48 collectors, maximum: 6 recruits). Most recruits (∼98% on average) were retrieved on the other components of the collectors (plastic container, plastic ring, plastic tube and sides/undersides of basalt rocks). The frequency of presence of recruits (at least one) on the top surfaces of each type of collector was more closely related to their abundance on the other components of the collectors than surface type on which they settled (Fig. 4B). The density of *P. resedaeformis* recruits was significantly greater on all other components of the collectors than on their flat surfaces at both walls of the canyon (paired *t*-tests; north wall – basalt rocks: *t*_9_ = 10.32, *P*<0.001; mesh pads: *t*_5_ = 6.19, *P* = 0.002; south wall - basalt rocks: *t*_9_ = 8.90, *P*<0.001; mesh pads: *t*_5_ = 4.75, *P* = 0.005), and for basalt rocks at the deepest location (floor: *t*_9_ = 3.30, *P* = 0.009).

**Figure 5:**
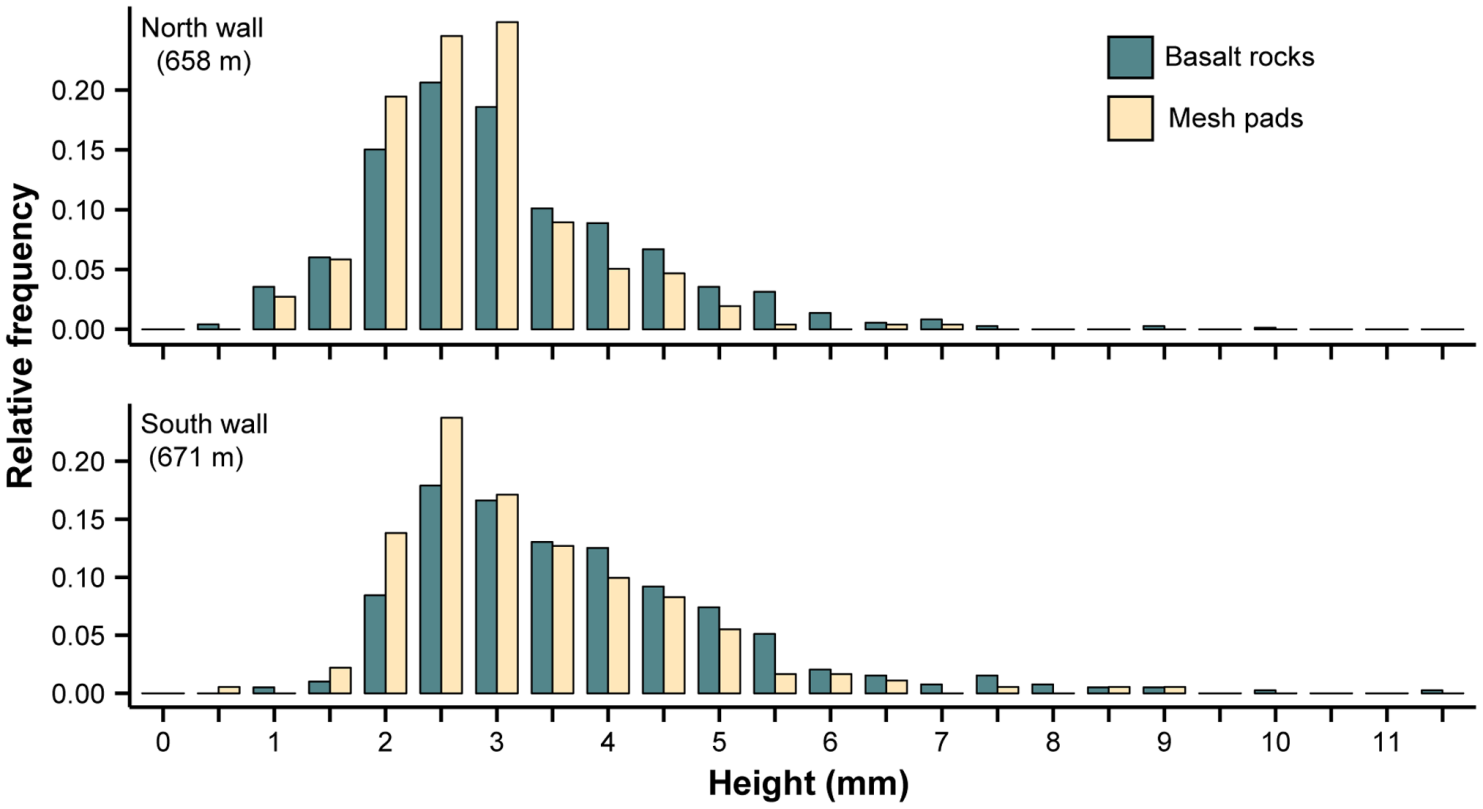
Relative height-frequency distributions of pooled *P. resedaeformis* recruits. Recruits were retrieved on larval settlement collectors with basalt rocks (north wall: n = 732; south wall: n = 391) and mesh pads (north wall: n = 257, south wall: n = 181). Heights indicate the lower ends of 0.5 mm-bins.

The height of *P. resedaeformis* primary polyps ranged from 0.85 mm to 9.30 mm (mean ± SD: 3.03 mm±0.99 mm; n = 1262), while the height of juvenile colonies ranged from 2.35 mm to 11.90 mm (4.78 mm±1.36 mm; n = 378). Relative height-frequency distributions of the *P. resedaeformis* recruits were unimodal and right-skewed at the south wall and north wall sites (Fig. 5), suggesting continuous recruitment. We compared the height-frequency distributions of recruits between the north and south walls, and between surface types within each location. Low recruitment restricted us from including the deepest location (floor) in this analysis. For both surface types, recruits were taller on the south wall (2-sample K-S test; basalt rocks: D = 0.20, *P*<0.001; mesh pads: D = 0.22, *P*<0.001) than the north, and within each of these locations, recruits were taller on basalt rocks than mesh pads (2-sample K-S test; south wall: D = 0.15, *P* = 0.005; north wall: D = 0.16, *P*<0.001).

**Figure 6:**
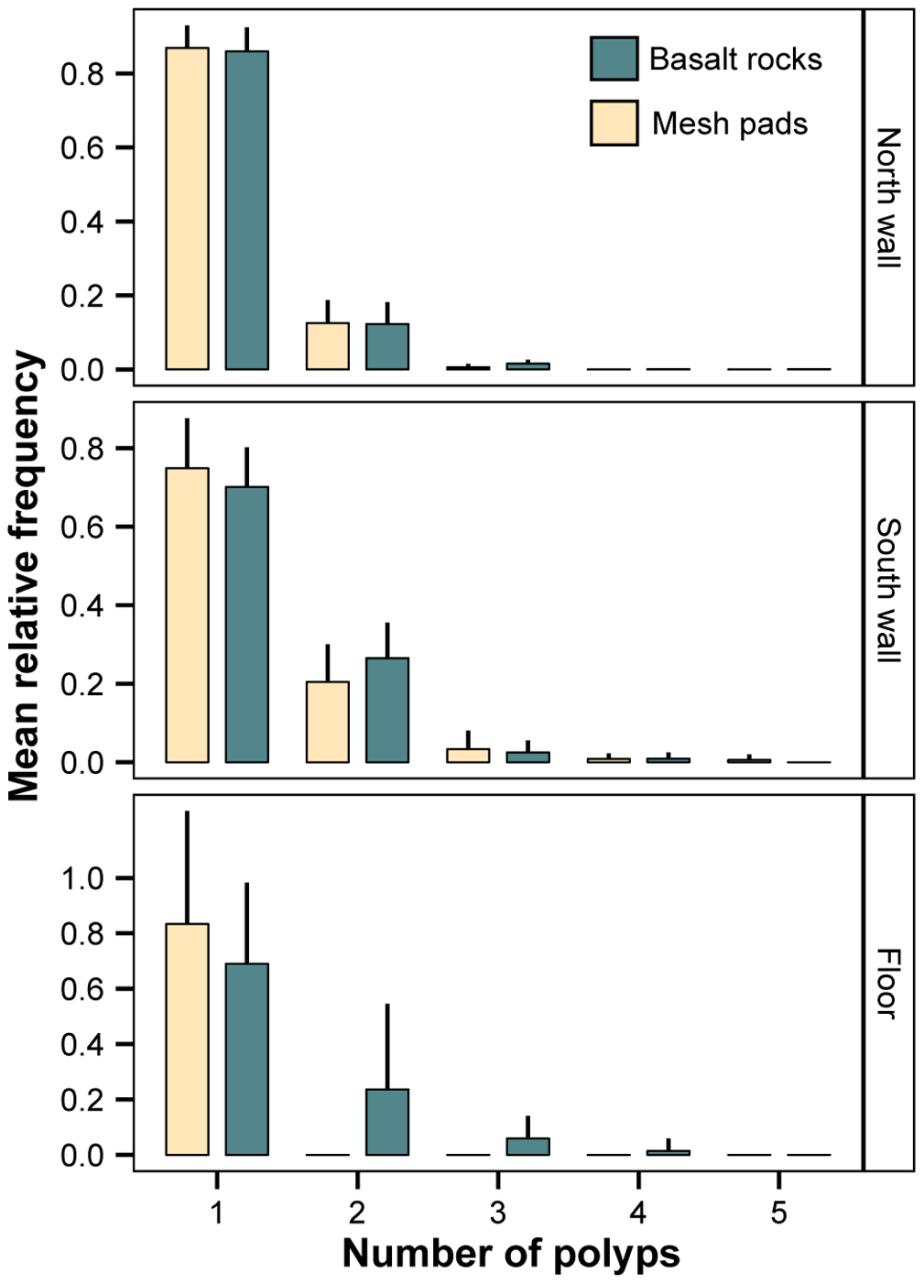
Mean relative frequency (+SD) of *P. resedaeformis* recruits on settlement collectors. = 10) or mesh pads (n = 6) on arrays deployed at 3 locations (north wall: 658 m, south wall: 671 m, floor: 863 m). The number of polyps reflects early life stages of the recruits: ‘one polyp’ is the primary polyp, and ‘2 or more polyps’ are juvenile colonies.

We consider each additional polyp on *P. resedaeformis* recruits as an important transition in the early development stages. The mean relative frequency of recruits decreased with increasing number of polyps, a pattern that was consistent on both walls and at the floor of the canyon, and on both surface types (Fig. 6). The majority of recruits were at the primary polyp stage (mean relative frequencies ranging from 70% to 86%), and on average, fewer than 10% of the recruits were found with 3 polyps or more. Recruits reached a maximum size of 5 polyps, found on the south and north walls of the canyon, and in both cases, represented fewer than 0.5% of the recruits from these locations.

## Discussion

In our study, recruitment on the collectors was high for *P. resedaeformis* (most likely reflecting significant larval supply for this species), but limited for *P. arborea*. Such differences could possibly be explained by divergent reproductive modes between the 2 species. Recruitment of corals depends on local larval availability, which itself is linked to the density of adults, their reproductive output (fecundity), and the dispersal potential of larvae [39].

Broadcast spawning is present in deep-water reef-building (scleractinian) corals [40], but studies on deep-water soft corals have generally reported gonochoric brooding as the main reproductive strategy [41]–[44]. A recent study based on fecundity analyses proposed that *P. resedaeformis* may be one of the few deep-water soft coral species to be a gonochoric broadcast spawner [45]. In our study, the abundance of *P. resedaeformis* recruits was higher on the north (depth: 658 m) and south (depth: 671 m) walls than on the floor (depth: 863 m) of the canyon, which reflected both the local abundance of colonies and general bathymetric range of this species. In the Middle Canyon of the Northeast Channel, the abundance of *P. resedeaformis* colonies peaks at depths between ∼ 500 and 650 m, decreasing at greater depths, and being virtually absent at depths greater than 750–800 m [33]. In this area, the distribution of colonies is clustered and dense thickets are frequently observed [33]. On the north wall, the maximum density observed was of 37.7 colonies/10 m^2^ (at depths of 500–525 m), while on the south wall, the maximum density reached 18.3 colonies/10 m^2^ (at depths of 600–625 m) [33]. *P. resedaeformis* recruits were overall more abundant, but shorter on the north wall, while they were less abundant, but taller on the south wall. This suggests asynchrony in recruitment dynamics among these locations, despite being at similar depths, and the potential importance of local conditions in influencing recruitment. However, recruits of *P. resedaeformis* were retrieved at the floor of the Middle Canyon, indicating a potential for this species to supply larvae beyond its reported range. In shallow waters, it is typically assumed that reef-building broadcast spawners disperse over wider areas than brooding species [46], but this relationship remains unclear [47]. The positively-buoyant eggs of spawners, followed by a generally longer larval pre-competency period than brooders, allow them to use wide-reaching ocean currents for dispersal [48]. The deep flow in Northeast Channel is dominated by tides [36], but local water circulation in the Middle Canyon is unknown. In general, submarine canyons have been reported to funnel water from the continental shelf to the deep sea known as ‘dense shelf water cascading’ [49]. Our results suggest that larvae of *P. resedaeformis* may be utilizing these currents, but the lack of suitable substrate (mostly comprised of coarse sand on the floor of the canyon, which is not the preferred substrate of this species [13], [15], [33], [38]) may prevent recruitment at great depths.

On the north and south walls, the magnitude of recruitment suggests that post-recruitment processes influence the local abundance of adult colonies. Such processes have been reported to be important in regulating the populations of shallow-water broadcast spawning corals [50]. Based on the mean abundance of recruits on the top surfaces of the collectors on the north wall (0.57 recruits/100 cm^2^) and south wall (1.42 recruits/100 cm^2^), estimated abundance of adult colonies would range between 5,700 and 14,200 colonies per 10 m^2^, 3.5 orders of magnitude greater than the measured mean abundance of colonies at these depths (2.7 to 4.1 colonies per 10 m^2^– [33]). Additionally, the majority of recruits were at the primary polyp stage, with a sharp decline in abundance in later stages. Given that the shape of the height-frequency distributions of *P. resedaeformis* recruits suggests continuous recruitment, in accordance with a previous study [45], we consider it unlikely that recent, large pulses in recruitment are the sources of these patterns at both locations.

The influence of post-recruitment processes on the local abundance of adult colonies is unknown in deep-water gorgonian corals. In our study, structural complexity influenced recruitment of *P. resedaeformis*, as larvae most often settled on other components of the collectors, rather than on the top flat surfaces. We suggest that survival of coral recruits may have been the result of the presence of refuges from biological disturbance. It is possible that *P. resedaeformis* is unable to recruit (despite high larval supply) on available suitable substrate (i.e. pebbles, cobbles and boulders) due to the presence of other benthic organisms creating a disturbance and affecting survival in the early life history of this species. In Northeast Channel, the density of the brittle star *Ophiacantha abbysicola* has been reported at more than 1,000 individuals/m^2^ [51]. These suspension-feeding brittle stars were abundant at the north and south wall sites in the vicinity of the collectors, and on the collectors when we retrieved them. They were relatively more abundant on the north wall than on the south wall, which could explain why more recruits were retrieved on the top flat surfaces of the collectors on the south wall (1.42 recruits/100 cm^2^ compared to 0.57 recruits/100 cm^2^ on the north wall). Further, recruits on basalt rocks were relatively larger than those on mesh pads, possibly because the space between the basalt rock and the plastic container provided a refuge from such disturbance.

The 3-dimensional structure of the collectors may have also altered fine-scale flow, possibly enhancing the survival of coral recruits by altering the encounter rate with food particles and reducing sedimentation. In dynamic deep-water habitats, food supply is through the export of surface primary production, presumably through vertical deposition and lateral advection in areas of strong currents. The presence of rich megafaunal communities in deep-water canyons [52] and on seamounts [53] suggests that food delivery is enhanced in these habitats, but the magnitude of this flux is unknown. At spatial scales of 10 s to 100 s of km’s, food supply is considered the dominant factor influencing the distribution of *P. resedaeformis* and *P. arborea* [12], [13], [15]. At scales of meters to 10 s of meters, in both moderate and steep reliefs, the abundance of these gorgonian corals is greater in the presence of structural complexity (cobbles, pebbles, and boulders) [15], which can enhance the resuspension of organic matter by creating turbulent flow near the seafloor [13]. Enhanced recruitment of suspension-feeding invertebrates in cryptic microhabitats, such as cracks and undersides of plates, rather than due to surface composition (basalt rock vs. plastics), has also been reported near hydrothermal vents [25]. Strong currents and turbidity near the seafloor concurrently reduce sedimentation, which can affect the suspension-feeding capacity of gorgonian corals. Encounter rate with food particles may hence not be sufficient to ensure the development of each primary polyp into a juvenile colony. The rates of export of primary production to the benthos off the continental margin of Atlantic Canada are not currently known. Such knowledge is urgently needed, as the dynamics of surface primary productivity are shifting in various water bodies worldwide, potentially affecting deep-dwelling suspension-feeders [54].

Unlike *P. resedaeformis*, the reproductive strategy of *P. arborea* has not been studied to date. *P. arborea* is typically larger than *P. resedaeformis*, forming a concave shape oriented perpendicular to the dominant direction of currents [8]. This species is thought to feed primarily on fresh phytodetritus [55], which could explain their shape to maximize the encounter rate with food particles in areas of strong currents, and their local distribution (they are often found on vertical structures - [13], [15], [33]). The abundance of *P. arborea* colonies does not follow a clear bathymetric pattern, but this species tends to be found at greater depths than *P. resedaeformis* [13], [15], [16], [33], [38]. In our study, the largest recruit (>20 polyps) was identified as *P. arborea*, and was retrieved at the floor of the canyon. It was the only location where 1) a large boulder (>3 m in diameter) was present, and 2) colonies of *P. arborea* were present within meters of the collectors, including one exceeding 1.5 m in height. It is possible therefore that the floor of the canyon was more susceptible to recruitment of *P. arborea*. Further, we consider it unlikely that the lack of *P. arborea* recruits may be due to space competition, given the high abundance of *P. resedaeformis* recruits and because much of the space available on the collectors had not been colonized. Given the important difference in recruitment between *P. arborea* and *P. resedaeformis*, and the presumed limited range of settlement for *P. arborea* (brooders release competent larvae, which can substantially reduce time spent in the water column [18]), we suggest that this species may be a brooder.

Overall, we conclude that both *P. resedaeformis* and *P. arborea* are limited in their ability to maintain their populations in the Northeast Channel Coral Conservation Area, although the reason differs between the 2 species. Deep-water gorgonian corals are long-lived invertebrates (10 s to 100 s of years), and processes affecting early life stages must be integrated over a longer period of time than for shallow-water species. After 4 years, we found very few recruits of *P. arborea*, which suggests low larval supply in the area. In contrast, despite high recruitment for *P. resedaeformis*, our results suggest extremely high mortality for this species in its early life stages: approximately 20% of the coral recruits formed a colony, and <1% of these were at a stage of ≥4 polyps. We emphasize that our study provides indirect evidence supporting hypotheses on the reproductive strategy of each species (*P. resedaeformis* as a broadcast spawner and *P. arborea* as a brooder), and that more information on the reproductive biology of each species is needed. We have also shown that recruitment is enhanced by the structural complexity of the recipient habitat, and proposed biological disturbance and access to limited food resources in the water column as ecological mechanisms possibly explaining this enhanced recruitment and high juvenile mortality early on in the benthic stage.
